# Long-Chain Fatty Acids and Inflammatory Markers Coaccumulate in the Skeletal Muscle of Sarcopenic Old Rats

**DOI:** 10.1155/2019/9140789

**Published:** 2019-07-01

**Authors:** Thea Laurentius, Robert Kob, Claudia Fellner, Mahtab Nourbakhsh, Thomas Bertsch, Cornel Christian Sieber, Leo Cornelius Bollheimer

**Affiliations:** ^1^Department of Geriatric Medicine, RWTH Aachen University Hospital, Germany; ^2^Institute for Biomedicine of Aging, Friedrich-Alexander-Universität Erlangen-Nürnberg, Nuremberg, Germany; ^3^Institute of Radiology, University Hospital Regensburg, Germany; ^4^Institute of Clinical Chemistry, Laboratory Medicine and Transfusion Medicine, General Hospital Nuremberg, Paracelsus Medical University, Nuremberg, Germany

## Abstract

Obesity and inflammation are reportedly associated with the pathogenesis of sarcopenia, which is characterized by age-related loss of skeletal muscle mass. Intramuscular fat deposits have been found to compromise muscle integrity; however, the relevant fat compounds and their roles as mediators of muscular inflammation are not known. The aim of this study was to identify potential correlations between inflammation markers and lipid compounds that accumulate in the quadriceps muscle of previously described Sprague-Dawley (SD) rat model for high-fat diet- (HFD-) induced muscle loss. Six-month-old SD rats were continuously fed a control (CD) or HFD until the age of 21 months. Magnetic resonance imaging (MRI) revealed a significant decline in muscle cross-sectional area in male SD rats as a result of HFD, but not in female rats. Here, we developed a new procedure to quantitatively identify and classify the fatty acid methyl esters (FAMEs) in rats' quadriceps muscles from our former study using gas chromatography-mass spectrometry (GC-MS). Fatty acid analysis revealed accumulation of octadecadienoic (linoleic acid), octadecanoic (stearic acid), and octadecenoic (vaccenic acid) acids exclusively in the quadriceps muscles of male rats. The designated fatty acids were mainly incorporated into triacylglycerols (TAGs) or free fatty acids (FFAs), and their proportions were significantly elevated by consumption of a HFD. Furthermore, the number of resident immune cells and the levels of the chemokines RANTES, MCP-1, and MIP-2 were significantly increased in quadriceps muscle tissue of HFD-fed male, but not female rats. Together, HFD-induced muscle loss in aged male SD rats is associated with greater deposits of long-chain fatty acid esters and increased levels of the inflammatory markers RANTES, MCP-1, and MIP-2 in skeletal muscle tissue. This trend is further reinforced by long-term consumption of a HFD, which may provoke synergistic crosstalk between long-chain fatty acids and inflammatory pathways in sarcopenic muscle.

## 1. Introduction

Sarcopenia was initially defined as degenerative loss of skeletal muscle mass and function as a result of aging [1]. Several epidemiological studies reported on the association between obesity and impaired muscle strength, but clinical studies on the combined effect of muscle impairment and obesity revealed controversial results, possibly due to different operational definitions [2]. Nevertheless, aging and obesity induce substantial changes in fat metabolism, increasing fat deposits in nonadipose tissue, such as skeletal muscles [3, 4]. Animal studies have revealed that increased intramuscular lipid accumulation may compromise muscle protein anabolism through lipotoxicity, leading to cellular dysfunction or induction of low-grade inflammation [5, 6]. Indeed, fatty acids can directly induce cytokine gene expression in cell culture experiments, and the relative potency of different fatty acid derivatives varies according to their chemical characteristics [7].

Chemokines are a family of approximately 50 small cytokines that induce directed chemotaxis in responsive cells [8]. The two main chemokine families are named according to their arrangement of N-terminal cysteine residues, CC or CXC chemokines. Together with approximately 20 different chemokine receptors, they form a complex chemokine system that orchestrates and fine-tunes the extent and dynamics of inflammation.

Lipids and their fatty acid compositions have been identified as indicators of age and nutritional status in humans, primates, and rodents [9]. Lipids are categorized into four major ester-lipid classes: cholesterol esters (CEs), triacylglycerols (TAGs), glycerophospholipids (GPLs), and free fatty acids (FFAs). Fatty acids are generally classified by chain length and saturation. Short-chain, medium-chain, and long-chain fatty acids encompass aliphatic tails of up to 6, 12, and 18 carbon atoms, respectively. Depending on their saturation, fatty acids are divided into saturated fatty acids (SFAs) and mono- (MUFAs) or polyunsaturated fatty acids (PUFAs). Conventional lipid analysis involves a chemical transesterification step to form fatty acid methyl esters (FAMEs) for further fatty acid characterization. FAMEs incorporate discrete classes of fatty acids, which have previously been involved in distinct cellular signaling pathways, like inflammation and lipotoxicity [7, 10, 11].

Magnetic resonance (MR) imaging has been widely used to study muscular changes in subjects with sarcopenia [12–14]. Changes in water organization and compartmentalization within muscle tissue are monitored by T2 relaxation time, a time constant characterizing the spin-spin signal decay in MR analyses [15]. Prolonged T2 relaxation times indicate impairments in skeletal muscle quantity and function [16]. Using this technique, we described the suitability of Sprague-Dawley (SD) rats as an experimental model of diet- and sex-related sarcopenia [17]. For instance, a high-fat diet (HFD) induces degenerative loss of skeletal muscle mass in SD rats compared with a control diet (CD) [13]. This degenerative process was exclusively observed in male rats and accompanied by an increase in caspase-3 activity, which is associated with proteolytic degradation of cytoskeletal proteins in muscle tissue [17]. Gender-specific regulation of fat metabolism has been well established, particularly in humans and rodents [18, 19]. Moreover, these sex-specific differences have also been observed with changes in physiological parameters such as body weight and plasma lipids. However, epidemiological data for sex-specific prevalence of sarcopenia have been conflicting [20–22]. Here, using gas chromatography-mass spectrometry (GC-MS) analysis, we further characterize the fatty acid species that accumulated in the quadriceps of the previously reported SD rats. Furthermore, analysis of inflammatory markers showed parallels between long-chain fatty acids and accumulation of Rantes (CCL5), MCP-1 (CCL2), and MIP-2 (CXCL2) chemokines in the sarcopenic muscle tissue of male SD rats.

## 2. Material and Methods

### 2.1. Animal Procedures

SD rats were obtained from Janvier Labs (Saint-Berthevin Cedex, France). Six-month-old SD rats were fed either a HFD (43 energy percent of neutral fat, based on lard and corn oil) or a CD (25 energy percent of neutral fat) until the age of 21 months. Compared with the CD (C1090_10, Altromin Spezialfutter GmbH & Co. KG, Lage, Germany), the HFD (C1090_45, Altromin Spezialfutter GmbH & Co. KG, Lage, Germany) contained twofold excess levels of all fatty acid species, and polysaccharides were reduced by 37% in compensation. The HFD resulted in a 14% increase in metabolic energy compared with the CD. The concentrations of all other components including proteins were equal in the HFD and CD. All rats were given ad libitum access to water and food and were housed in groups of 3 rats per cage at a constant room temperature of 20°C and on a 12-hour light-dark cycle until the end of the experiment. Animals' daily energy intake from CD and HFD was controlled to be equal throughout the study (Supplementary Materials, S1). A number of animals were excluded based on injuries or early development of tumors [23]. The final study included 12 HFD-fed (6 male and 6 female) and 17 CD-fed (12 male and 5 female) rats. The protocol was approved by the Committee on the Ethics of Animal Experiments of the University of Regensburg, Germany.

### 2.2. Magnetic Resonance Imaging and Spectroscopy (MRI and MRS) Examination

SD rats were anesthetized by an intraperitoneal injection of pentobarbital (Narcoren®, Merial, Hallbergmoos, Germany), and oxygen was delivered via a mask during subsequent examinations. Proton magnetic resonance spectroscopy (^1^H-MRS) was performed. MRI and 1H-MRS were performed with a 1.5-Tesla clinical scanner (Magnetom Avanto; Siemens Healthcare, Erlangen, Germany) using an 8-channel array coil designed for human knee examinations. T1-weighted spin echo (SE) sequences were applied to image the quadriceps muscles of extended fore limbs. MR spectra were obtained from the right and left *musculus vastus lateralis* (voxel size 12 × 6 × 7 mm^3^). The relative lipid content was calculated as the lipid fraction = [MRS_lipids_/(MRS_lipids_ + MRS_water_)] × 100. The values for MRS _water_, which represents the field under the water signal in the spectrum recorded without water suppression, and MRS _lipids_, the measurement under the lipid signals, ranged from 0.9 to 1.6 ppm and from 1.9 to 2.6 ppm, respectively. The T2 maps across the quadriceps muscle sections were obtained using a multiecho spin sequence (12 echoes, 14–169 ms).

### 2.3. Preparation of Lipid Fractions from Rat Quadriceps Muscles

Immediately after MRS examination, all animals were sacrificed by decapitation. Absolute intramuscular tissue sections from the right and left *musculus vastus lateralis* were isolated and stored at -80°C. Next, 50 mg of muscle tissue was homogenized in PBS (1 : 10, *v*/*v*). For analysis of the CE, TAG, and GPL fractions, 500 *μ*L of tert-butyl methyl ether (90%, tert-BME) and 250 *μ*L of methanol were added to 500 *μ*L of each homogenized muscle tissue sample. The mixtures were then vortexed for 1 min and centrifuged at 10,000×g for 1 min. The upper tert-BME layer containing total lipids was collected and used in further preparations. For analysis of the FFA fractions, 1 mL of a chloroform/methanol (2 : 1) mixture was added to 500 *μ*L of each homogenized muscle tissue sample.

The isolation of CE, TAG, and GPL lipid classes was performed using a method similar to a previously described protocol for blood samples [24]. Dry silica gel solid phase extraction (SPE) columns (200 mg of silica gel) were each loaded with 100 *μ*L of the collected tert-BME layers and washed with 20 *μ*L of hexane. Columns were incubated at room temperature (RT) for 5 min and dried in a vacuum overnight (at least 5 hours). The processing methods are described below. The CE fraction was eluted using 3.4 mL of 1% methyl acetate/hexane (*v*/*v*). Next, the TAG fraction was eluted using 3 mL of 2.5% methyl acetate/hexane (*v*/*v*). The column was then washed with 3 mL of acetone. Next, the GPL fraction was eluted using 4 mL of methanol.

For isolation of FFAs, dry silica gel SPE columns (200 mg of silica gel) were each loaded with 100 *μ*L of the collected chloroform layers. The FFA fraction was eluted with 3 mL of 80% hexane/diethyl ether (*v*/*v*) and 4 mL of methanol. The eluates were mixed with 2 mL 1% sulfuric acid in methanol and incubated at 50°C for one hour with mixing every 15 min. The mixture was incubated at RT for additional 10 min and supplemented with 3 mL of 5% sodium chloride. The hexane layer was collected and washed twice with 3 mL of water.

### 2.4. Transesterification and Preparation of FAMEs from Different Lipid Classes

To process the CE fraction, 1.5 mL of acetone and 600 *μ*L of a 2 M sodium methoxide solution (CH_3_ONa) were added, and the mixture was vortexed and incubated at RT for 30 min. 100 *μ*L of acetic acid and 3 mL of water were added to each sample. The hexane layer was collected and washed twice with 3 mL of water and loaded onto an SPE column. The resulting FAMEs from the CE fraction were eluted with 3 mL of 1% methyl acetate-hexane (*v*/*v*).

To process the TAG fraction, 100 *μ*L of acetone and 100 *μ*L of 2 M CH_3_ONa were added, and the mixture was incubated at RT for 30 min. 20 *μ*L of acetic acid and 3 mL of water were added, and the mixture was vortexed again. Next, the hexane layer was collected and washed three times with 3 mL of water and loaded onto an SPE column. The resulting FAMEs from the TAG fraction were eluted with 3 mL of 1% methyl acetate/hexane (*v*/*v*).

To process the GPL fraction, 600 *μ*L of a 2 M CH_3_ONa was added, and the mixture was vortexed and incubated at RT for 7 min. 100 *μ*L of acetic acid, 3 mL of hexane, and 3 mL of water were added, and the mixture was vortexed again. Next, the hexane layer was collected and washed using 3 mL of water and loaded onto an SPE column. The resulting FAMEs from the GPL fraction were eluted with 3 mL of methyl 1% acetate/hexane (*v*/*v*).

To further process the FFA fraction, FFA fraction in hexane was loaded onto an SPE column. The resulting FAMEs were eluted with 3 mL of 1% methyl acetate/hexane (*v*/*v*).

### 2.5. Gas Chromatography-Mass Spectrometry (GC-MS)

FAMEs were detected using a 6890N gas chromatograph (Agilent Technologies, Santa Clara, California, USA) equipped with a flame ionization detector coupled to a 5973N quadrupole mass spectrometer. One microliter of each sample was injected with a split ratio of 1 : 20 at 250°C. A BPX-70 column with an internal diameter (ID) of 50 m × 0.22 mm and film thickness of 0.25 *μ*m (SGE Analytical Science, Austin, Texas, USA) was utilized with a helium flow rate of 1.4 mL/min. The oven temperature was continuously increased from 145°C to 240°C at a constant rate of 3°C/min for separation. Then, substances were identified using a quadrupole mass spectrometer (Agilent Technologies, Santa Clara, California, USA). The mass spectrometer source and transfer line were set to 250°C in N_2_ gas.

### 2.6. Histology

Muscles were fixed with 4% paraformaldehyde and embedded in paraffin. Sections with a thickness of 5 *μ*m were stained with Masson's trichrome (Sigma-Aldrich, St. Louis, Missouri, USA) according to the manufacturers' instructions. Among the myofibers (bright pink with violet nuclei), immune cells emerged with dark blue-green cytoplasm and brown nuclei. The number of immune cells was determined using direct microscopic examination of 10 randomly selected fields of 0.1 cm^2^ in muscle sections from all study animals.

### 2.7. Multiplex Immunoassay

A ProcartaPlex immunoassay (Thermo Fisher Scientific, Waltham, Massachusetts, USA) was used to assess the levels of 22 rat inflammatory markers (IL-1 alpha, G-CSF, IL-10, IL-17A, IL-1 beta, IL-6, TNF alpha, IL-4, GM-CSF, IFN gamma, IL-2, IL-5, IL-13, IL-12p70, Eotaxin, GRO alpha, IP-10, MCP-1, MCP-3, MIP-1 alpha, MIP-2, and Rantes) in single muscle tissue samples. Sample preparation, assays, and analyses were performed as described in the manufacturer's instructions.

### 2.8. Statistical Analysis

The data are presented as the means ± standard deviation (SD). Two-way ANOVA was applied to analyze and compare data between the groups as specified in the figure legends. The Bonferroni method was employed to correct for multiple comparisons. Differences with *p* values ≤ 0.05 (after Bonferroni correction) were considered statistically significant in all experiments.

## 3. Results

### 3.1. SD Rats as a Model for Diet-Induced Sarcopenia

**Figure 1:**
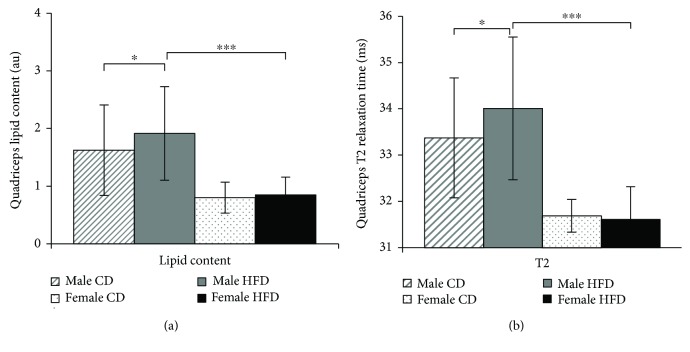
Correlation between lipid contents and T2 relaxation times in the quadriceps muscles of aging SD rats. (a) Lipid contents in the right and left quadriceps muscles of 21-month-old rats are shown as the means ± SD of independent animals. (b) Maximum cross-sectional T2 relaxation times of the right and left quadriceps muscles of 21-month-old rats are shown as the means ± SD of 12 CD-fed and 6 HFD-fed males (dashed and solid gray bars, respectively), as well as 5 CD-fed and 6 HFD-fed females (dotted and solid black bars, respectively). Statistical significance was determined using two-way ANOVA and is denoted by ^∗^ for *p* ≤ 0.01, ^∗∗^ for *p* ≤ 0.005, and ^∗∗∗^ for *p* ≤ 0.001.

In a previous study, we examined 12 HFD-fed (6 male and 6 female) and 17 CD-fed (12 male and 5 female) healthy Sprague-Dawley (SD) rats and found that feeding of high-fat diet (HFD) consistently led to weight increase in male and female rats [13, 17]. Female rats demonstrated successive loss of skeletal muscle cross-sectional area through aging. However, male rats demonstrated a more substantial loss in the skeletal muscle cross-sectional area, which further declined as a result of continuous HFD consumption [13, 17]. Based on this model of sarcopenia, we hypothesized that the loss of skeletal muscle mass in obese male SD rats may be caused by selective accumulation of distinct lipid derivatives. In the current study, we used the quadriceps muscle tissues from SD rats in our former study to perform a detailed lipid analysis. For a better comparison, the total lipid concentrations that accumulated in the quadriceps muscles of 21-month-old male and female SD rats are presented in Figure 1(a). The data show that the quadriceps muscles of male SD rats accumulated 97.3% (*p* ≤ 0.05) more lipids than those of female rats. Moreover, HFD-fed male rats accumulated 17% (*p* ≤ 0.01) more lipids in the quadriceps muscles than CD-fed male rats. These trends were further confirmed by comparison of quadriceps T2 relaxation times as a measure of muscle tissue integrity [16]. Compared with male rats, the T2 relaxation times in female rats were markedly lower independent of dietary fat. In contrast, T2 relaxation times were 2.1 ms (*p* ≤ 0.05) longer in the quadriceps of male rats and further extended by 0.7 ms (*p* ≤ 0.001) as a result of HFD consumption (Figure 1(b)).

### 3.2. Fatty Acid Profile in the Quadriceps Muscles of SD Rats

**Figure 2:**
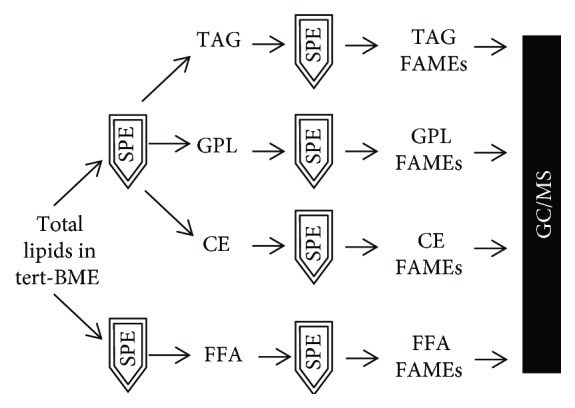
Schematic of the SPE procedure for GC-MS analysis of FAMEs from rat tissue or plasma. Total lipid fractions from plasma or tissue were separated on 2 SPE columns to isolate a fraction of FFAs or another fraction containing CEs, TAGs, and GPLs, respectively. For each fraction, a second SPE column was used to purify the individual FFA, CE, TAG, or GPL fractions for further GC-MS analysis.

All rats were euthanized and the quadriceps muscles were isolated immediately. The quadriceps muscles of the male SD rats were used for a more in-depth biochemical and histological study of sarcopenia. Muscle tissue extracts were subjected to two parallel silica gel SPE steps in order to obtain a fraction of free fatty acids (FFAs) and another fraction of O-ester-lipid classes containing cholesterol esters (CEs), triacylglycerols (TAGs), and glycerophospholipids (GPLs). Each of the FFA, CE, TAG, and GPL fractions was transesterified to FAMEs and further purified using a second SPE step (Figure 2). In addition, equal amounts of reference FAMEs were added to lipid fractions for validation of the extraction procedure and the GC-MS analysis. The quantities of fatty acids from different lipid classes were calculated as a percentage of the total amounts of FAMEs in the respective sample.

**Figure 3:**
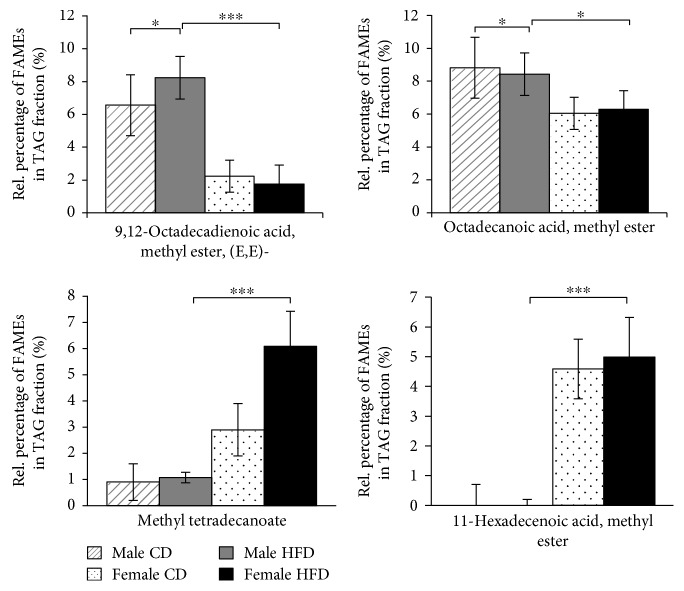
Accumulation of FAMEs in the TAG fractions from quadriceps muscles of 21-month-old SD rats. Diagrams show the percentage of each indicated FAME (*x*-axis) among the total TAG fractions from 21-month-old rats as the means ± SD of 12 CD-fed and 6 HFD-fed males (hatched and solid gray bars, respectively), as well as 5 CD-fed and 6 HFD-fed females (dotted and solid black bars, respectively). Statistical significance was determined using two-way ANOVA and is denoted by ^∗^ for *p* ≤ 0.01, ^∗∗^ for *p* ≤ 0.005, and ^∗∗∗^ for *p* ≤ 0.001.

**Figure 4:**
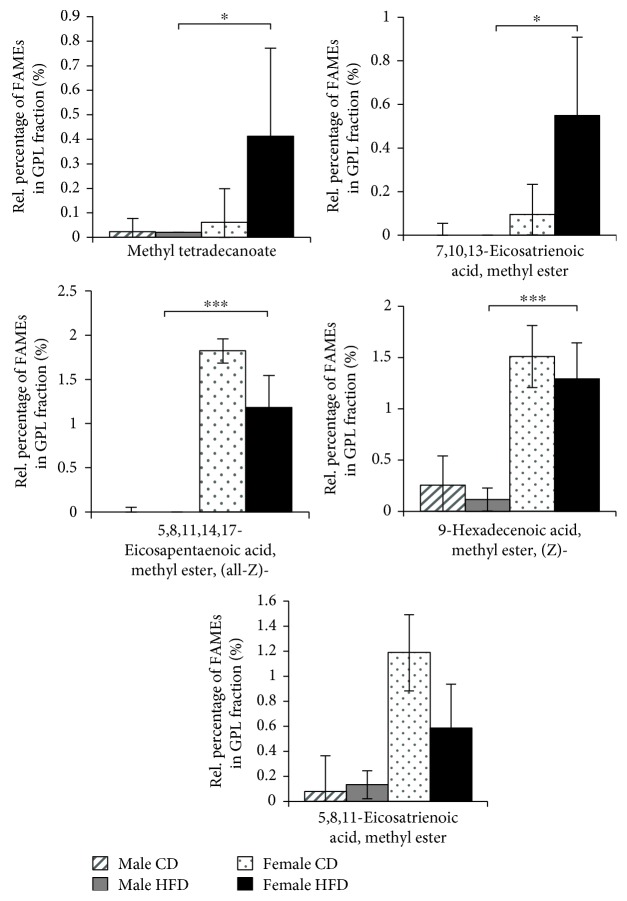
Accumulation of FAMEs in the GPL fractions from quadriceps muscles of 21-month-old SD rats. Diagrams show the percentage of each indicated FAME (*x*-axis) among the total GPL fractions from 21-month-old rats as the means ± SD of 12 CD-fed and 6 HFD-fed males (hatched and solid gray bars, respectively), as well as 5 CD-fed and 6 HFD-fed females (dotted and solid black bars, respectively). Statistical significance was determined using two-way ANOVA and is denoted by ^∗^ for *p* ≤ 0.01 and ^∗∗^ for *p* ≤ 0.005.

**Figure 5:**
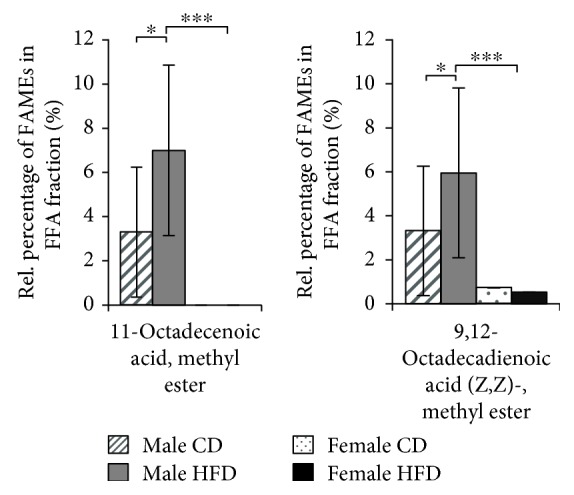
Accumulation of FAMEs in the FFA fractions from quadriceps muscles of 21-month-old SD rats. Diagrams show the percentage of each indicated FAME (*x*-axis) among the total FFA fractions from 21-month-old rats as the means ± SD of 12 CD-fed and 6 HFD-fed males (hatched and solid gray bars, respectively), as well as 5 CD-fed and 6 HFD-fed females (dotted and solid black bars, respectively). Statistical significance was determined using two-way ANOVA and is denoted by ^∗^ for *p* ≤ 0.01.

We identified a total of 25 different fatty acids in the SD rat quadriceps muscle (Table 1). Fifteen different fatty acid species were detected in all samples, regardless of sex or diet regime, confirming the consistency of the analytical procedure (Supplementary Materials, S2). Ten fatty acid species were differentially represented in TAG, GPL, and FFA fractions from quadriceps muscles of female and male rats (Figures 3, 4, and 5). In the TAG fractions, higher levels of octadecanoic acid (stearic acid), methyl ester and 9,12-octadecadienoic acid (linoleic acid), methyl ester (E,E)- were detected in the quadriceps muscles of male rats, whereas significantly higher levels of methyl tetradecanoate (myristic acid) and 11-hexadecenoic acid (lycopodic acid), methyl ester were detected in the quadriceps muscles of female rats (Figure 3). Interestingly, all other fatty acids were predominantly present in GPL fractions from the quadriceps muscles of female rats (Figure 4) or in FFA fractions from the quadriceps muscles of male rats (Figure 5). In addition to the sex-related disparities, we observed distinct effects of different diet regimes. For instance, the amount of 9,12-octadecadienoic acid (linoleic acid), methyl ester (E,E)- in TAGs was increased by 21% (*p* ≤ 0.01) and the amounts of 11-octadecenoic acid (vaccenic acid) and 9,12-octadecadienoic acid (Z,Z)- (linoleic acid), methyl esters in FFA by 95% (*p* ≤ 0.05) in HFD-fed male rats (Figures 3 and 5). Thus, male SD rats exhibit a compelling predisposition to accumulation of long-chain fatty acid esters in their skeletal muscles, which is further enhanced by long-term HFD feeding.

### 3.3. Inflammatory Status in the Quadriceps Muscles of SD Rats

**Figure 6:**
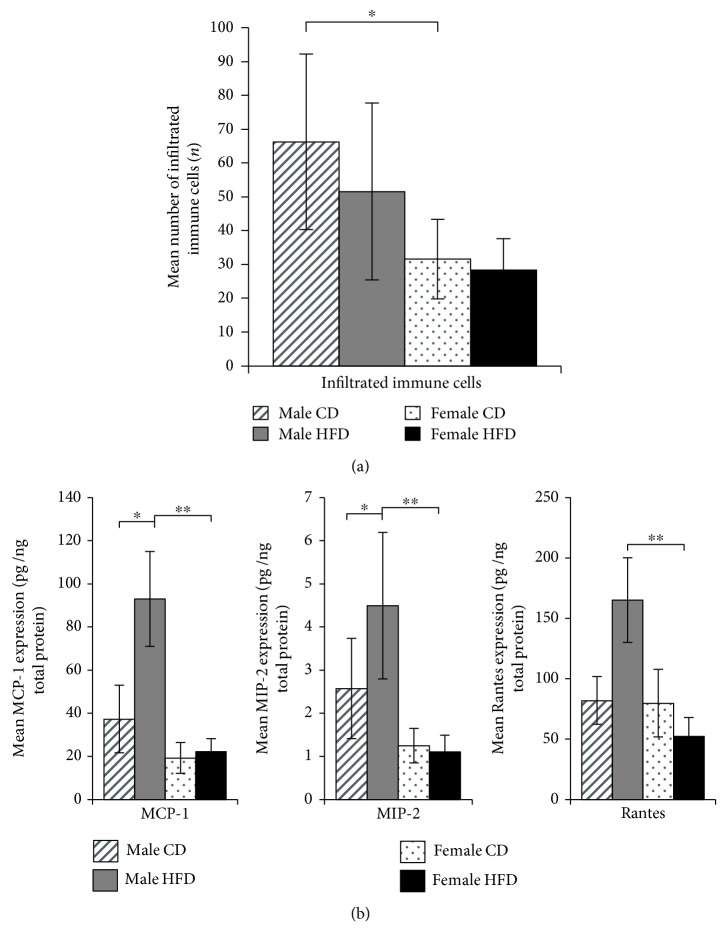
(a) Resident immune cells in the quadriceps muscles of 21-month-old SD rats. The diagram shows the number of resident immune cells determined by direct microscopy of 10 randomly selected microscopic fields of Masson's trichrome-stained muscle sections of all study animals. Bars indicate the mean numbers of cells ± SD from 12 CD-fed and 6 HFD-fed males (hatched and solid gray bars), as well as 5 CD-fed and 6 HFD-fed females (dotted and solid black bars, respectively). Statistical significance was determined using two-way ANOVA and is denoted by ^∗^ for *p* ≤ 0.01. (b) Chemokine expression in the quadriceps muscles of 21-month-old SD rats. The diagram shows the concentration of each indicated chemokine (*x*-axis) in 1 ng total tissue extract from 21-month-old rats as the means ± SD of 12 CD-fed and 6 HFD-fed males (hatched and solid gray bars, respectively), as well as 5 CD-fed and 6 HFD-fed females (dotted and solid black bars, respectively). Statistical significance was determined using two-way ANOVA and is denoted by ^∗^ for *p* ≤ 0.01 and ^∗∗^ for *p* ≤ 0.005.

In addition to the proposed impact of dietary fat on systemic inflammation, various fatty acids have been reported to directly stimulate innate immune cells through Toll-like receptors [7, 11]. We first examined the total number of resident immune cells in muscle tissue sections from SD rats. The quadriceps muscles of male SD rats contained more immune cells than those of female rats, whereas immune cell numbers were not significantly affected by diet (Figure 6(a)). Next, the levels of 22 inflammatory markers were simultaneously detected in equal amounts of total extracts from the quadriceps muscle tissues of SD rats. Out of 22, the levels of 19 chemokines were not affected by sex or diet (Supplementary Materials, S3). Only the levels of three chemokines, Rantes, MCP-1, and MIP-2, were differentially regulated in the skeletal muscle of male SD rats by the HFD (Figure 6(b)). The expression of MCP-1 and MIP-2 was approximately 60-70% (*p* ≤ 0.05) higher in CD-fed males compared to females. Moreover, the consumption of the HFD led to a 72.2%, 80.3%, and 62.8% (*p* ≤ 0.05) increase in MCP-1, Rantes, and MIP-2 expression in quadriceps muscles of male SD rats, respectively (Figure 6(b)).

## 4. Discussion

The main findings of this study were that long-term consumption of HFD favors the accumulation of distinct long-chain fatty acid esters as well as Rantes and MCP-1 chemokines in skeletal muscle tissue of male SD rats compared to female SD rats and that this effect may contribute to increased loss of muscle mass. In our previous study, we reported a gradual apoptotic loss of skeletal muscle tissue in HFD-fed Sprague-Dawley (SD) rats; however, the components that may link fat metabolism to sarcopenia remained unknown [17]. This current report verifies the utility of the SD rat model for studies of sarcopenia using two distinguished criteria, prevalent lipid accumulation and longer T2 relaxation times of the quadriceps muscles. Consistent with the results from the SD rat model, several human studies have revealed a higher prevalence of sarcopenia in men than in women [25, 26]. However, the prevalence of sarcopenia may vary depending on age and the diagnosis criteria. For instance, Dam et al. reported a higher prevalence in women using the criteria of the Foundation for the National Institutes of Health (FNIH) [27].

Nevertheless, SD rats represent a sex-discriminating model of sarcopenia that enabled the identification of lipid species and inflammatory markers, which are associated with substantial loss of muscle mass exclusively in male rats. Notably, the majority of the analyzed lipid species and chemokines did not vary by sex or diet (Supplementary Materials, S2 and S3). This uniformity was highly valuable to confirm the accuracy of the study design and performance, but it also emphasized the relevance of the anticipated sex- and diet-related differences.

### 4.1. Prevalence of Unsaturated Long-Chain Fatty Acids in Sarcopenic Muscle Tissue

The accumulation of fatty acids in the skeletal muscle has been attributed to ectopic lipid deposition during HFD-induced obesity [28, 29]. This accumulation may result from enhanced lipid uptake or decreased lipid use through oxidation, lipid release, and/or secretion. In the present study, we precisely monitored the food lipid quality and intake without any distinguishable sex-based variations (data not shown). Thus, male and female rats likely processed the provided food differently.

Female SD rats accumulated more unsaturated short-chain fatty acids as triacylglycerols (TAGs) or glycerophospholipids (GPLs). The levels of some of the accumulated fatty acids were further increased by the HFD, suggesting both diet- and sex-dependent regulation of lipid deposition in the skeletal muscle tissue of female rats. In comparison, the skeletal muscle of sarcopenic male rats accumulated greater amounts of long-chain fatty acids incorporated into TAG and free fatty acid (FFA) fractions. The most significant increases were observed in the FFA levels (Table 2). This increase was even more evident in the HFD-fed animals. In fact, the amount of monounsaturated 11-octadecenoic acid (vaccenic acid), methyl ester in the female quadriceps samples was extremely low, approximately 366.46-fold lower than in the CD-fed male and 777.53-fold in the HFD-fed male quadriceps. We speculate that unsaturated long-chain octadecenoic and octadecadienoic acids are to some extent relevant to the loss of muscle mass and strength in male SD rats.

Unsaturated long-chain fatty acids are absorbed at a higher rate than saturated fatty acids of similar chain lengths [30]. This phenomenon has been attributed to differences in the binding affinity of fatty acids to cytosolic fatty acid-binding proteins (FABPs) [31]. However, unsaturated long-chain fatty acids are most susceptible to free radical damage [32]. Thus, long-chain fatty acids act as paracrine factors that potentially modulate the mitochondrial beta-oxidation capacity [33]. In our previous study, we did not observe any differences in the anabolic Akt or ubiquitin ligase pathways in the muscle tissues of male or female SD rats [17]. However, caspase-3-dependent apoptosis was shown to be involved in the successive decrease in muscle mass in aging male rats but not in female rats [5, 17].

### 4.2. Inflammatory Profile of Sarcopenic Muscle Tissue

The population of resident immune cells in the skeletal muscle increases with aging, and they can potentially secrete numerous regulatory factors that are candidate mediators of skeletal muscle regeneration [34]. We observed a greater number of resident immune cells in the skeletal muscle of aged male SD rats, consistent with the sex-discriminating sarcopenia model. Although the immune cells were not further characterized here, their total number was not further increased by long-term HFD feeding. Thus, sex and diet may drive discrete regulatory mechanisms during the development of sarcopenia. This hypothesis was further supported by a distinguished pattern of intramuscular chemokine expression. Long-term HFD feeding mediated a significant increase in the levels of Rantes, MCP-1, and MIP-2, which did not exhibit significant variations between CD-fed male and female rats. Notably, the levels of 19 other chemokines were not affected by sex or diet (Supplementary Materials, S3).

According to previous *in vitro* studies, fatty acid supplements directly induce cytokine gene expression in cultured immune cells [7, 11, 35]. Saturated short-chain fatty acids revealed the greatest activity, but unsaturated fatty acids failed to activate an inflammatory response in these studies [7, 11, 35]. In contrast, our data confirm a simultaneous increase in the levels of 3 different inflammatory mediators and long-chain unsaturated fatty acids in the skeletal muscle of HFD-fed sarcopenic rats. This discrepancy may be partially explained by the use of contrasting *in vivo* and *in vitro* study approaches. On the other hand, metabolite-sensing long-chain fatty acid receptors, such as GPR43 and GPR120, likely play important roles in this process. Although these receptors are expressed on immune cells, their possible involvement in the activation of muscle-resident immune cells remains elusive [36].

Diet-induced obesity was previously shown to restrain regeneration and repair processes in the skeletal muscle tissue of mice [37, 38]. Our present data imply an important role of MCP-1, Rantes, and MIP-2 in this process, which may result from the accumulation of long-chain fatty acids in the skeletal muscle of sarcopenic SD rats. MCP-1 is chemotactic to monocytes and macrophages, which are associated with ongoing inflammation, alterations in glucose metabolism, and insulin resistance [39, 40]. Rantes is chemotactic to basophil cells and disturbs skeletal muscle regeneration in mice [41]. MIP-2 is secreted by monocytes and macrophages and chemotactic to leukocytes and hematopoietic stem cells. Although the total number of intramuscular immune cells was not significantly increased in the skeletal muscle of male SD rats in this study, the simultaneous expression of MCP-1, Rantes, and MIP-2 can contribute to the accumulation of a specific subpopulation of immune cells and a sustained inflammatory response.

## 5. Conclusions

Sarcopenia is strongly associated with a predominant accumulation of inflammatory markers and long-chain unsaturated free fatty acids in muscle tissue resulting from enhanced lipid uptake, decreased oxidation, or both. Further studies are required in the future to distinguish between these possibilities and to determine the utility of these potential biomarkers in patient specimens. Nonetheless, the medical significance of lipid metabolism, specifically the accumulation of unsaturated long-chain FAMEs in muscle tissue, may serve as an early indicator of sarcopenia.

## Figures and Tables

**Table 1:** Detected FAMEs in the quadriceps muscles of aging SD rats.

TAG	Methyl tetradecanoate
	Pentadecanoic acid, methyl ester
	Hexadecanoic acid, methyl ester
	9-Hexadecenoic acid, methyl ester, (Z)-
	11-Hexadecenoic acid, methyl ester
	Octadecanoic acid, methyl ester
	9-Octadecenoic acid, methyl ester, (E)-
	11-Octadecenoic acid, methyl ester
	9,12-Octadecadienoic acid, methyl ester, (E,E)-

GPL	Methyl tetradecanoate
	Hexadecanoic acid, methyl ester
	9-Hexadecenoic acid, methyl ester, (Z)-
	Octadecanoic acid, methyl ester
	9-Octadecenoic acid, methyl ester, (E)-
	11-Octadecenoic acid, methyl ester
	9,12-Octadecadienoic acid, methyl ester
	5,8,11-Eicosatrienoic acid, methyl ester
	7,10,13-Eicosatrienoic acid, methyl ester
	5,8,11,14-Eicosatetraenoic acid, ethyl ester, (all-Z)-
	5,8,11,14,17-Eicosapentaenoic acid, methyl ester, (all-Z)-
	4,7,10,13,16,19-Docosahexaenoic acid, methyl ester, (all-Z)-

CE	Hexadecanoic acid, methyl ester
	Octadecanoic acid, methyl ester
	9-Octadecenoic acid (Z)-, methyl ester

FFA	Methyl tetradecanoate
	Hexadecanoic acid, methyl ester
	9-Hexadecenoic acid, methyl ester, (Z)-
	Heptadecanoic acid, methyl ester
	Octadecanoic acid, methyl ester
	9-Octadecenoic acid, methyl ester
	11-Octadecenoic acid, methyl ester
	9,12-Octadecadienoic acid (Z,Z)-, methyl ester
	5,8,11,14-Eicosatetraenoic acid, methyl ester, (all-Z)-
	4,7,10,13,16,19-Docosahexaenoic acid, methyl ester, (all-Z)-

**Table 2:** Prevalent FAMEs in the quadriceps muscles of aging SD rats presented as the male to female ratio of FAME levels in the rats.

Lipid class	FAMEs	Male/female ratio	
		CD	HFD
TAG	9,12-Octadecadienoic acid, methyl ester, (E,E)-	2.94	4.67
	Octadecanoic acid, methyl ester	1.46	1.34

FFA	11-Octadecenoic acid, methyl ester	**366.46**	**777.53**
	9,12-Octadecadienoic acid (Z,Z)-, methyl ester	**4.62**	**11.25**

## Data Availability

The data used to support the findings of this study are available from the corresponding author upon request.

## Supplementary Materials

Supplementary 1 S1: daily energy intake of male SD rats. Two to three male SD rats were housed in each cage. We recorded the food intake multiple times during the study. The diet intake in each cage was calculated as *W* = initial food weight–leftover food weight–spilled food weight. The daily energy intake per rat (kJ/day/rat) was calculated as *C* − *W* ^∗^total energy of the chow diet. HFD-fed rats consumed only ~1% more total calories than CD-fed rats throughout the entire study (*p* = 0.21, two-way *t*-tests).

Supplementary 2 S2: accumulation of FAMEs in the quadriceps muscles of 21-month-old SD rats. Diagrams show the percentage of each indicated FAME (*x*-axis) among the total TAG (A), GPL (B), and FFA (C) fractions as the mean ± SD of 12 CD-fed and 6 HFD-fed males (hatched and solid gray bars, respectively), as well as 5 CD-fed and 6 HFD-fed females (dotted and solid black bars, respectively). ANOVA revealed no statistical significance.

Supplementary 3 S3: chemokine expression in the quadriceps muscles of 21-month-old SD rats. The diagram shows the concentration of each indicated chemokine (*x*-axis) in 1 ng total tissue extract from 21-month-old rats as the mean ± SD of 12 CD-fed and 6 HFD-fed males (hatched and solid gray bars, respectively), as well as 5 CD-fed and 6 HFD-fed females (dotted and solid black bars, respectively). ANOVA revealed no statistical significance.
