# Supplementary material for: Long-Chain Fatty Acids and Inflammatory Markers Coaccumulate in the Skeletal Muscle of Sarcopenic Old Rats
Source: Dis Markers. 2019 Jul 1;2019:9140789. doi: 10.1155/2019/9140789 (PMC6636585; doi:10.1155/2019/9140789)
Supplement: Supplementary file 1 [file 9140789.f1.docx]

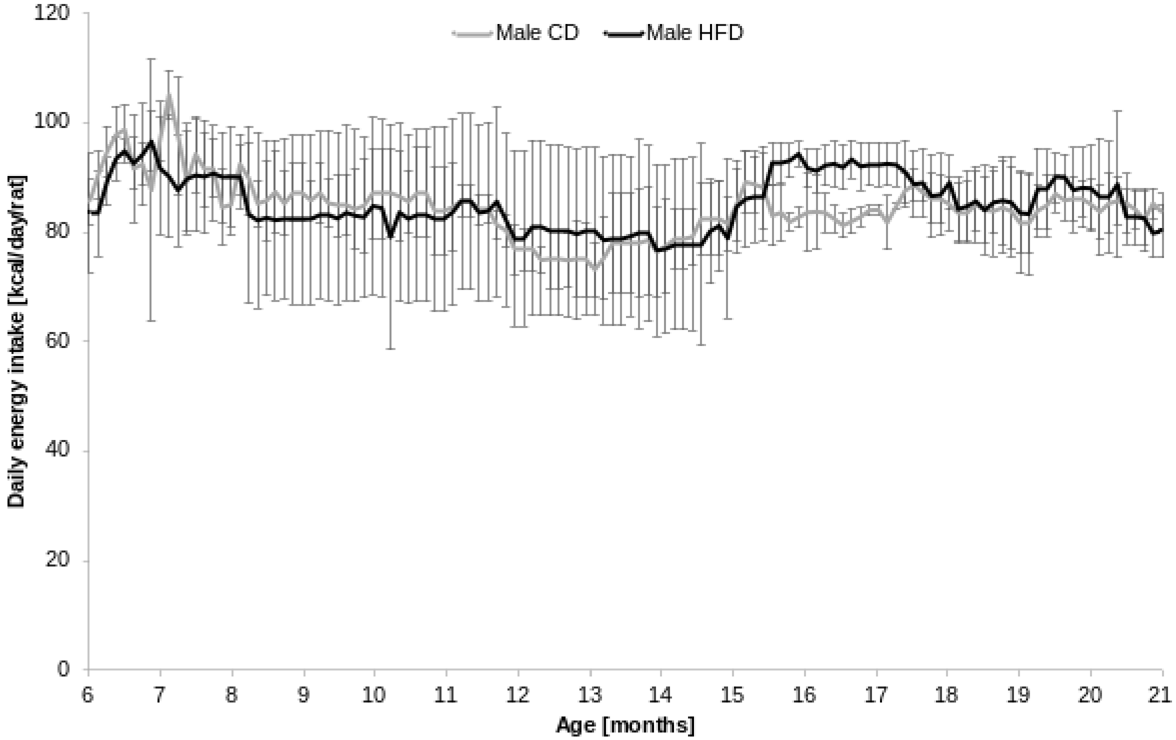

S1. Daily energy intake of male SD rats. Two to three male SD rats were housed in each cage. We recorded the food intake multiple times during the study. The diet intake in each cage was calculated as W = initial food weight–leftover food weight–spilled food weight. The daily energy intake per rat (kJ/day/rat) was calculated as: C-W*total energy of the chow diet. HFD-fed rats consumed only ~ 1 % more total calories than CD-fed rats throughout the entire study (p = 0.21, two-way T-tests).

Supplementary Material , Laurentius et al. 2019
