# Supplementary material for: Long-Chain Fatty Acids and Inflammatory Markers Coaccumulate in the Skeletal Muscle of Sarcopenic Old Rats
Source: Dis Markers. 2019 Jul 1;2019:9140789. doi: 10.1155/2019/9140789 (PMC6636585; doi:10.1155/2019/9140789)
Supplement: Supplementary file 2 [file 9140789.f2.docx]

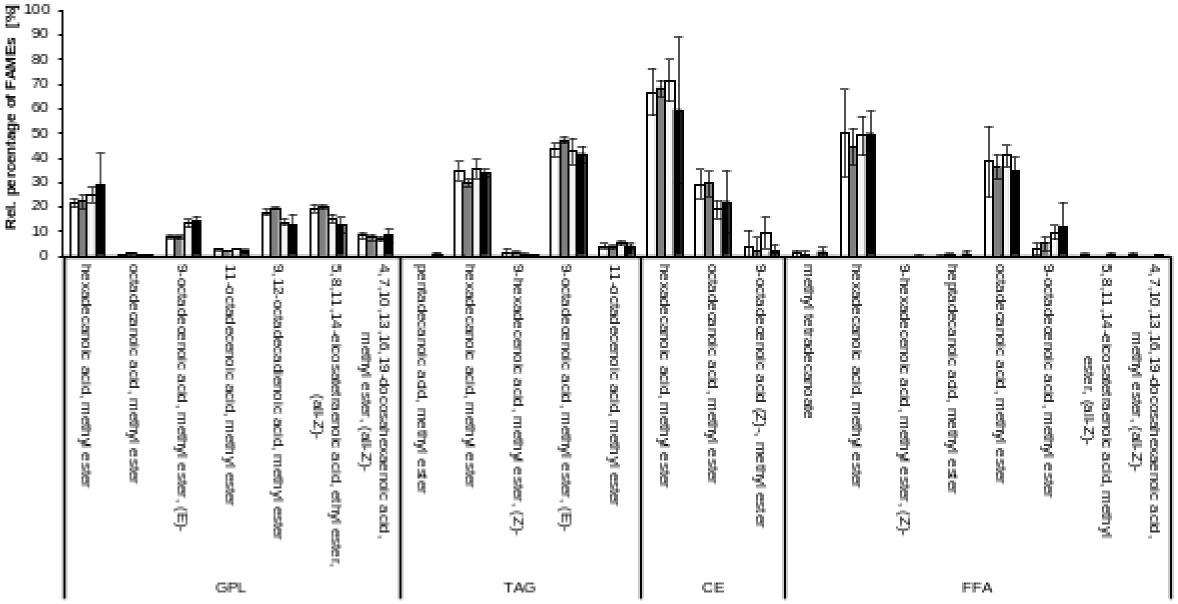

S2. Accumulation of FAMEs in the quadriceps muscles of 21-month-old SD rats. Diagrams shows the percentage of each indicated FAME (x-axis) among the total TAGs (A), GPLs (B) and FFAs (C) fractions as the mean SD of 12 CD-fed and 6 HFD-fed males (hatched and solid gray bars, respectively), as well as 5 CD-fed and 6 HFD-fed females (dotted and solid black bars, respectively). ANOVA revealed no statistical significance.
