# Supplementary material for: Long-Chain Fatty Acids and Inflammatory Markers Coaccumulate in the Skeletal Muscle of Sarcopenic Old Rats
Source: Dis Markers. 2019 Jul 1;2019:9140789. doi: 10.1155/2019/9140789 (PMC6636585; doi:10.1155/2019/9140789)
Supplement: Supplementary file 3 [file 9140789.f3.docx]

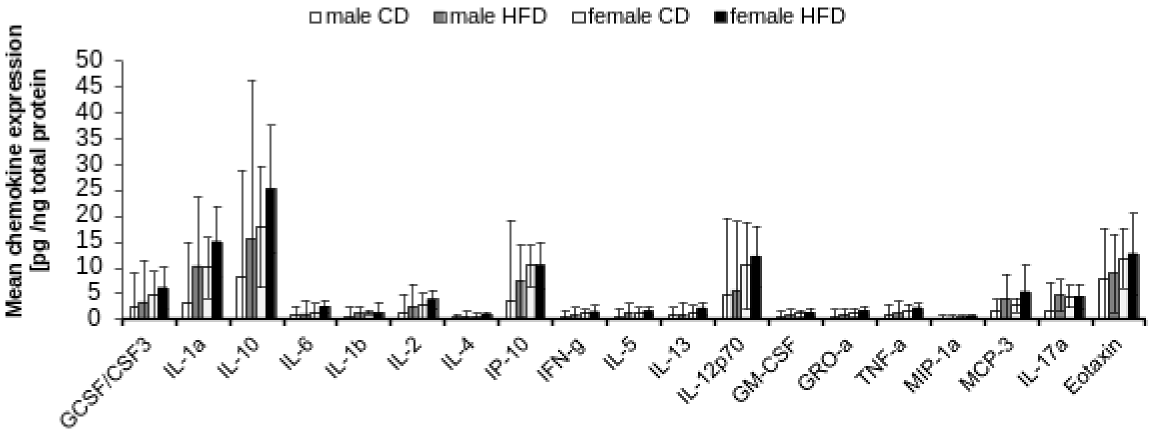

S3. Chemokine expression in the quadriceps muscles of 21-month-old SD rats. The diagram shows the concentration of each indicated chemokine (x-axis) in 1 *n*g total tissue extract from 21-month-old rats as the mean SD of 12 CD-fed and 6 HFD-fed males (hatched and solid gray bars, respectively), as well as 5 CD-fed and 6 HFD-fed females (dotted and solid black bars, respectively). ANOVA revealed no statistical significance.
